# Case of Epilepsy

**Published:** 1827-12

**Authors:** 


*Case of Epilepsy.* Detailed by the Patient.*

Sir,—I have for some years past been myself subject to this painful and dangerous complaint, and have taken much pains

* A very intelligent man, not a member of the profession.

to investigate its cause, and ascertain its symptoms. I have consulted many medical works upon the subject, as well as taken the opinion of medical men; but have never yet met, from one or the other, with a correct statement of the symptoms of the disorder. The description of them in both cases has been general, and such as would not inform a patient clearly whether he had epileptic fits or any other. These circumstances have induced me thus to publish my case, which I consider to be that of true and genuine epilepsy, for the use of the public at large, being thoroughly convinced that no person can so well describe the symptoms as those who, like myself, have had many attacks of it, and have attentively considered the preliminary sensations which indicate its approach.

The symptoms, as I have experienced them, are as follows:—First, sudden faintness comes on, attended by impeded respiration; a sense of tension and heat are felt on the left side of the stomach, just under the heart, which begins to beat with great violence, accompanied by an universal tremor. The right eye is then affected, and there is a defect of vision and great pain in the muscles of the eye; tears are forced out in considerable quantities from both eyes; the muscles of the right eye feel to be drawn to the right with great violence; and the vision is obscured by the light assuming a prismatic appearance, and, during the convulsion, a perfect rainbow is seen, which appears to ascend, and the eye mechanically seems to follow it. The left eye is then affected in the same manner, but not to quite so great a degree; and the muscles of the face, neck, arms, and legs, and lastly the whole body, become convulsed. The patient now falls down; the carotid artery, and the temporal arteries, throb with great force. The heart also flutters, and that with intermission; the pulsation at the wrist is scarcely felt, and sometimes is wholly suspended. The mind is filled with strange ideas of cataracts, voices, rattling of carriages, &c.; and there is great noise in the ears, arising, no doubt, from the impeded circulation of the blood through the carotid artery. The head now begins to turn round towards the right shoulder, whilst there is a sensation at the heart as if it were grasped in the hand. The eyes are violently convulsed, and the prismatic corruscations are more quick and vivid; the senses are obscured, and the breathing quick and laborious, till, in about half a minute after, the head becomes giddy, and animation (or at least consciousness) is suspended.

Till the head turns, I have not lost the power of thinking, and even asking advice, though this requires great exertion, owing to the convulsion of the mouth and throat. Having had many of these attacks, perhaps a dozen or more, I have become so perfectly acquainted with all the symptoms that occur, that I have divested my mind, by reflection, of any fear of them, and have in a few instances, once or twice, prevented their occurrence, so late as the first appearance of the corruscations, by immediately lying down on my back, and calming myself by an exertion of self-command, which, though difficult on account of fear, yet, when that is subdued by reflection, the mental operations can go on without interruption.

By doing this in time, (that is, two or three minutes after the first symptoms of faintness and impeded circulation at the heart,) and taking half a wine-glass of any spirit, I have found that the pulsation becomes more regular, the corruscations cease, and the fit is prevented. But there will still remain an unequal action of the pulse, a twitching of the tendons, a dulness and sense of weight and pain in the head, and a tendency to relapse, which requires great firmness of mind to resist. These symptoms will continue for three or four hours; and I have sometimes known them succeeded by a general shaking, like an ague, but without any sense of cold: on the contrary, there has been a glow all over the body.

The above prismatic corruscations take place at an angle with the axis of the eye, and the rays of light begin to oscillate as a perfect white light at first, of extreme brightness; they then become refracted, their corruscations increase in quickness, till they assume the most perfect and vivid appearance of the prismatic series.

The rationale of this phenomenon appears to be the convulsion that takes place in the muscles of the eye. The humors being thus thrown out of their proper form into various irregular figures by the convulsion, must naturally refract the rays of light, and thus form on the retina the prismatic spectrum, which is transmitted to the common sensorium; and this can only account for the appearance, or rather the fancied appearance, of the spectrum when the eyes are closed. I conceive it would be difficult, if not impossible, to produce this effect by artificial means, as it would be necessary to procure a natural eye, or gelatinous substances of the same qualities in every respect, and to give them, by galvanism or other means, the convulsive motions in precisely the same manner as in epileptic fits.

There is another singular affection to which I am sometimes subject, which is a quivering light, assuming during its continuance various figures, commencing at the exterior angle of the eyes, (the right beginning first,) and going off either over the eyebrow, or next the nose. All objects viewed at this time are obscured in part by the corruscations, and the effect produced is like that of looking through a pane of glass which by a defect in its manufacture is wavy.

This appears to be produced by a convulsion of the iris, which, rendering its contractions and irritations unequal, would naturally produce the effect described without any prismatic phenomenon.

